# Lectures on the Principles and Practice of Physic, Delivered at King’s College, London

**Published:** 1847-11

**Authors:** 


Art. IX.—*Lectures on the Principles and Practice of Physic, delivered at King's College, London.* By Thomas Watson, M.D., Fellow of the Royal College of Physicians; late Physician to the Middlesex Hospital, and formerly Fellow of St. John's College, Cambridge. Third American, from the last London edition. Revised, with Additions, by D. Francis Condie, M.D., Secretary of the College of Physicians; Author of a Treatise on the Diseases of Children, etc. Philadelphia: Lea and Blanchard. 1040 pp. 8vo. 1847.

Since the first reprint of this work was made in this country, a number of treatises on the Practice of Physic have appeared, but not one superior to it in merit. The high opinion of it expressed by the reviewers in London, on its first appearance, has been fully sustained by the American profession, and it stands now confessedly in the first rank of the publications relating to the practice of medicine. The editor has enhanced its value by his contributions of facts and observations furnished by his own and the experience of his brethren on this side of the Atlantic. Like all the issues of the enterprising house to which we are indebted for these Lectures, the volume in its mechanical execution is all that the reader could desire. Our thanks are due to Messrs. Maxwell and Prescott for their politeness in placing a copy of it on our table.

